# Characterization of Airborne Particles Collected from Car Engine Air Filters Using SEM and EDX Techniques

**DOI:** 10.3390/ijerph13100985

**Published:** 2016-10-01

**Authors:** Birmania Heredia Rivera, Martín Gerardo Rodriguez

**Affiliations:** Physiology and Pharmacology Department, Center of Basic Sciences, Autonomous University of Aguascalientes, Aguascalientes 20931, Mexico; herb62_@hotmail.com

**Keywords:** particle, pollen, SEM microscopy, car air filter

## Abstract

Particulate matter accumulated on car engine air-filters (CAFs) was examined in order to investigate the potential use of these devices as efficient samplers for collecting street level air that people are exposed to. The morphology, microstructure, and chemical composition of a variety of particles were studied using scanning electron microscopy (SEM) and energy-dispersive X-ray (EDX). The particulate matter accumulated by the CAFs was studied in two categories; the first was of removed particles by friction, and the second consisted of particles retained on the filters. Larger particles with a diameter of 74–10 µm were observed in the first category. In the second one, the detected particles had a diameter between 16 and 0.7 µm. These particles exhibited different morphologies and composition, indicating mostly a soil origin. The elemental composition revealed the presence of three groups: mineral (clay and asphalt), metallic (mainly Fe), and biological particles (vegetal and animal debris). The palynological analysis showed the presence of pollen grains associated with urban plants. These results suggest that CAFs capture a mixture of atmospheric particles, which can be analyzed in order to monitor urban air. Thus, the continuous availability of large numbers of filters and the retroactivity associated to the car routes suggest that these CAFs are very useful for studying the high traffic zones within a city.

## 1. Introduction

The composition of air, which is a mixture of solid particles, liquids, and gases, is important in determining life quality in big cities. Air pollution is a growing problem generated mainly by industrial and vehicular emissions. Particulate matter (PM) in urban areas is made up of dust deposited on the soil as well as by particles released by anthropogenic activities [1,2,3]. PM is usually characterized by its physical size or diameter and composition, ranging from nanometers (nm) to several tens of micrometers (µm). Particles with a diameter less than 10 μm are classified as PM_10_. Particles with diameters between PM_10_ and PM_2.5_ are defined as the coarse fraction [4,5]. PM_2.5_ includes all particles with diameters less than 2.5 μm, also known as fine particles. These fine particles are of special concern for two reasons. First, these particles are able to penetrate deep into the human respiratory system and can be absorbed into the blood, where they have been shown to cause biological effects. Second, these particles scatter light very efficiently and therefore play a major role in visibility impairment [6]. The smallest particles, ultrafines, have diameters <0.1 µm. Ultrafines, which are the most numerous but have the smallest mass and volume, are the subject of recent investigations into health effects [7]. Particle deposition in the various regions of human respiratory system depends strongly on particle size and shape by the complex action of aerosol deposition mechanisms [8], with the greatest fractional deposition occurring in the deep lung between 5 nm and 100 nm. The study of their composition is a powerful tool for evaluation of the effects of pollution on health and for identification of pollution sources [9].

Because of the diversity of sources, these particles vary greatly in their size, morphologies, and chemical composition, therefore, it is essential to understand the size distribution and chemical composition of the particles, particularly in the urban atmosphere. A detailed characterization of individual atmospheric particles also provides useful information about their sources, formation, reactivity, transport, and removal. Scanning electron microscopy (SEM) with energy-dispersive X-ray (EDX) analysis is commonly used for the study of single particles [10], as this method provides useful information on the morphology, elemental composition, and density of aerosols and also provides insight into the particle origin, which may be from anthropogenic or natural processes [11]. To date, PM_2.5_ is monitored by means of conventional sampling methods that include high-volume air sampling through a filter, and these methods have been used for decades to monitor atmospheric concentrations of PM. These systems use a pump to draw air through a glass fiber filter to collect particulate species, however, this type of sampling can be costly and is not always feasible. These stationary samplers are usually placed onsite where a power supply is necessary and cannot provide an overall air pollution level data for a large area, thus the determined air concentrations are somewhat site-specific.

Recently. Zhang et al. [12] and Katsoyiannis et al. [13] suggested that car air filters (CAFs) from taxis could act as “moving” high volume air samplers to provide city-integrated air concentrations with low variability for particle-bound contaminants. CAFs, which are necessary components of automobiles, are usually composed of polyurethane and other fiber-like materials, most frequently cotton, foam, or paper. The manufacturing process is accomplished using a pleated filter material and the addition of resins and glues. Paper air filters are widely used because these materials are disposable and inexpensive. The air filter set is usually placed inside a plastic or metal box connected to the throttle body with an intake tube. When the engine is working, air is taken into the cylinder and any dust in the air is retained on the CAF. Therefore, the CAFs have main functions of particle removal, engine protection, and maintaining performance at high levels. We have shown the usefulness of CAFs as samplers which can move a high volume of air, are inexpensive, and offer the possibility to determine the levels of air contaminants in downtown city streets. Their studies focused on the determination of pollutants in the CAFs; however, the filters retain dust, suggesting that these filters could be used to characterize the nature and composition of the PM trapped by urban automobiles filters. This idea represents a more realistic way of assessing the exposure of people to air pollution within the city. Therefore, the aims of this work were to estimate the size distribution and elemental composition of dust particles collected from the urban atmosphere by CAFs and to identify the main sources of street dust of Aguascalientes City in Mexico.

## 2. Materials and Methods

### 2.1. Study Area

**Figure 1:**
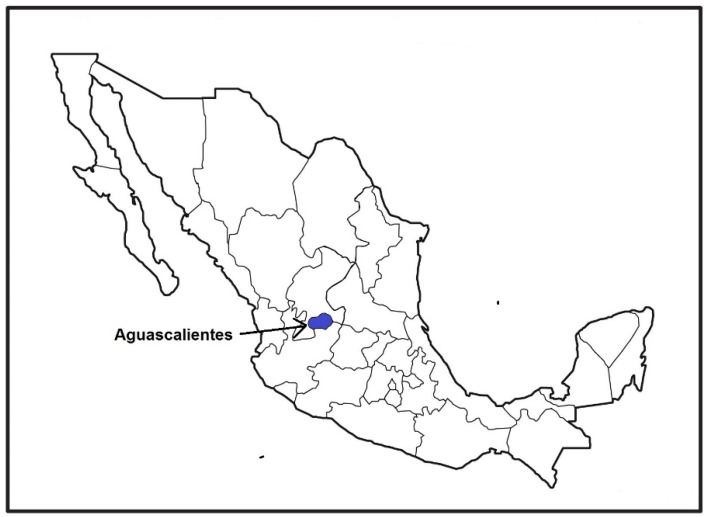
Map of Aguascalientes State.

The CAFs were collected from service agencies (Ford, Chevrolet, Toyota, and Nissan) located to the north of Aguascalientes City (21°53’ N, 102°18’ W). The CAFs were obtained from vehicles that circulate in the north of the city. This area is composed of main roads with six-lane highways, and four of these are considered to have high speeds and heavy traffic. These roads are paved with asphalt. Aguascalientes is the capital of the state of Aguascalientes, Mexico (Figure 1) and has an area of 385 km^2^, a population of 832,712 habitants, and more than 356,210 vehicles. Seasonal meteorological conditions at Aguascalientes include warm, dry weather, and the average annual rainfall ranges from 500 to 600 mm. This climate is characterized by evaporation that exceeds precipitation and is mainly associated with vegetative communities of desert-type scrub and vegetation, covering approximately 86.30% of the surface. The annual average temperature is 17.4 °C. The prevailing winds come from the southwest, but during the winter cold north winds and frosts often occur.

### 2.2. Sample Collection 

Emissions from local vehicles have been reduced to meet Profepa and Aguascalientes emission standards. The rules establish a scheduled maintenance once or twice annually for vehicle inspection to maintain the vehicle in normal operating conditions, however, although these standards establish the measurement of solid particle emissions, although this is rarely done in practice. Manufacturers of new cars in Mexico offer engine warranties if the buyer obtains the preventive maintenance service. The service agencies recommend changing CAFs between 6000 and 10,000 km. The role of CAFs, which are located between the air intake and the engine, is to protect the engine from air particulate matter. We only collected CAFs from vehicles that sought service at authorized agencies with an average record of 12,347 ± 664 km, from different brand vehicles but, 2014–2015 models. The collection time was between 6 January and 30 March, 2015. The sample consisted of removal of 10 CAFs directly from the plastic box connected to the throttle body with an intake duct. The CAFs were then placed in sterilized plastic bags by the technician and stored in the laboratory at 4 °C until analysis.

### 2.3. Methodology to Remove Particles from the CAFs.

The collected CAFs were reviewed to rule out any perforations. Physical dimensions (length, width, height, and space between sheets) were used to estimate the surface air intake. The particles collected by the filters were divided into two categories; the first consisted of those particles that could be removed by friction. The other one are those adhered particles which were not removed by friction.

**Figure 2:**
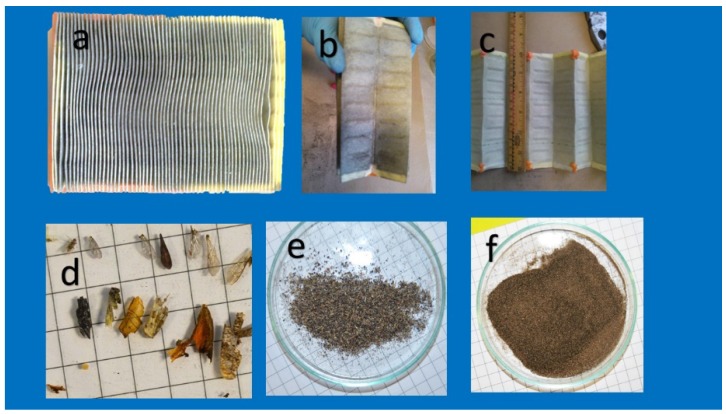
(**a)** Car air filter collected, (**b,c**) blends show the dust trapped, (**d–f**) macroparticles and dust retained.

The first group was separated by shaking the filter on a vortex plate, any extra material or particles on the filter were detached gently with a fine brush. Individual samples were over-dried at 40 °C and 20% relative humidity for 24 h, then sieved through a 200-mesh sieve to remove other oversize materials. The dust recovered was weighed using an analytical balance (Figure 2). After sieving, five fractions were obtained with a following particle diameter: 74–60 µm, 59–56 µm, 55–44 µm, 43–20 µm, and 19–10 µm. Until analysis, sub-samples were weighted and stored in polyethylene flasks in a cool and dry place. The separated particles were observed using transmission and reflected light microscopy (model illuminated with optic fiber ring and equipped with a Panasonic GP-KR222 analog camera (Panasonic ,Tokyo, Japan) and frame grabber (Encore Electronics, Los Angeles, CA, USA). Image analysis was performed using a micrometer rule (200 line/mm) and the Image J software information about the program can be found on http://rsbweb.nih.gov/ij/. To study the particles deposited on the CAFs, the cellulose filter was removed from the metallic and polyurethane foam supports, and small pieces of filter were studied by scanning electron microscopy.

### 2.4. Scanning Electron Microscopy and EDX from Particles Adhered on the CAFs

For SEM sample preparation, filter pieces of about 0.5 cm^2^ were cut with scissors from the center of each sample CAF, and mounted on 12.5 mm SEM stubs for gold coating. A very thin film of gold (Au) was deposited on the surface of each sample using a Gold Sputter Coater (Desk II) vacuum coating unit (Denton Vacuum LLC, Moorestown, NJ, USA). The SEM-EDX analysis of morphology and chemical composition of individual particles was carried out using a computer-controlled field emission SEM instrument (JSM-6330F, JEOL, Peabody, MA, USA). The EDX was carried out for each elemental analysis using the line scan analysis technique, and the present elements were both qualitative and quantitatively measured (Oxford INCA X-Act, Oxford Instruments, Buckinghamshire, UK). Particle counting was performed using the Image J software. Only particles larger than 0.2 µm were counted. For quantitative element analyses, EDX spectrograms were recorded and the weight percentage of each element present in the spectrum was identified. As mentioned above, the CAFs samples were gold coated. Therefore, the gold data of EDX cannot be used to estimate the quantitative elemental analyses, the Au contribution was manually subtracted during the evaluation of the EDX spectra. Three new CAFs that were purchased from official service agencies were used as controls and were prepared and analyzed identically to the experimental samples.

### 2.5. Biological Particle Analysis

Palynological analysis of the organic material trapped on the CAFs was performed according to the method used for melissopalynology. Briefly, 0.1 g of dust was placed into 10 mL test tubes and washed by centrifugation and decantation using distilled water. Then, a 10% potassium hydroxide solution was added to the residue, which was then warmed at 70 °C for 10 mins with occasional stirring. The material was filtered on a 300 µm filter, centrifuged twice for 5 min at 3000 rpm, and then decanted twice. The residue was treated with 7 mL acetolysis mixture (9:1 v:v acetic anhydride and concentrated sulfuric acid). The sample was warmed to 80 °C for 5–10 minutes with occasional stirring. Next the acetolysis mixture was removed by centrifugation and decantation. The residue was washed with ethyl alcohol and transferred to an Eppendorf tube. Two drops of glycerin were added, and the open tube was placed into an incubator at 40 °C for 12 h. The slides were then prepared using the method of Jones [14]. They were observed with light microscopy. Some positive samples were prepared in a similar way for scanning electron microscopy as described in the paragraph above.

## 3. Results

The analyses of the CAFs revealed that the average weight of the retained dust was 0.94 ± 0.72 g (n = 10). CAFs had a total filtration surface of 1.2 ± 0.5 m^2^, with a mean interspace between the sheets of 2.4 ± 1.23 mm, in agreement with data from the ISO 5011 test [15]. 

**Figure 3:**
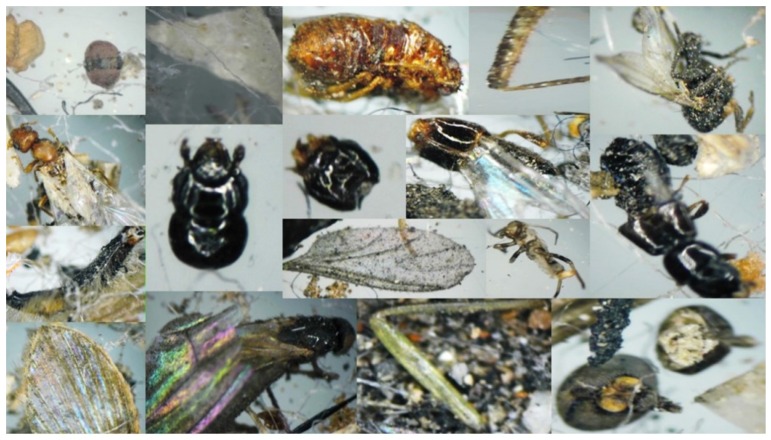
Mosaic of animal debris collected by the CAFs.

**Figure 4:**
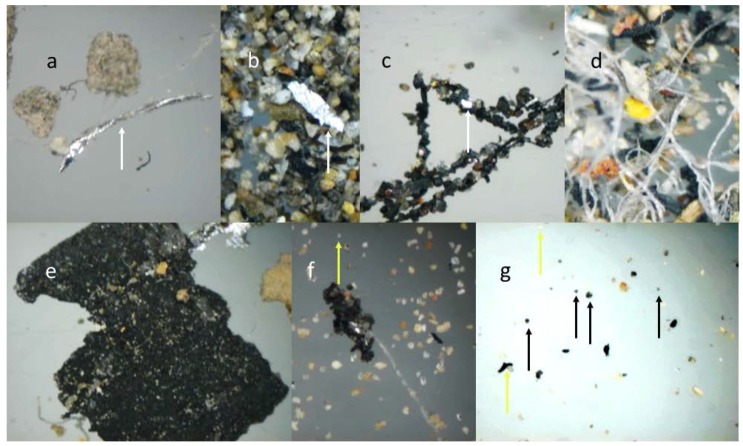
(**a–c**) Representative metallic and (**c–g**) inorganic particles observed by reflected light microscopy; (**d**) Synthetic fibers were also observed. Arrows on f and g indicate small particles.

The dust retained by CAFs has a heterogeneous composition due to its varied origin, transport, and emissions. Poschl [16] indicated that the particle sizes, chemical composition, and mixing states of atmospheric aerosols significantly impact climate and human health. The dust particles varied greatly with respect to size, morphologies, and chemical composition. Particles trapped in the 200 µm mesh were represented mainly by large biological material that was visible to the naked eye. Small insects, such as beetles, wasps, earwigs, bees, and flies, as well as legs, antennae, eyes, and wings were found (Figure 2 and Figure 3), and plant debris, represented by fragments of wood, seeds, and leaves, was also observed, albeit less frequently. Inorganic particles and synthetic fibers were also found (Figure 4). 

The observation of particles with reflected light microscopy allowed identification of the origin of removed particles, and many of these were biological in nature. Metallic particles observed under reflected light microscopy exhibited sizes ranging from 0.07 mm to 1.2 mm, with elongated shapes and shiny surfaces. Other material frequently present included natural and synthetic fibers characterized by transparency. The inorganic material was composed mainly of sands and small clays, which when viewed under a reflected light microscope were colorful (Figure 4a,b). These materials were a variety of shapes and sizes, conglomerates of sand with asphalt were observed in all CAFs and were identified as typical paving material (Figure 4e–g).

**Figure 5:**
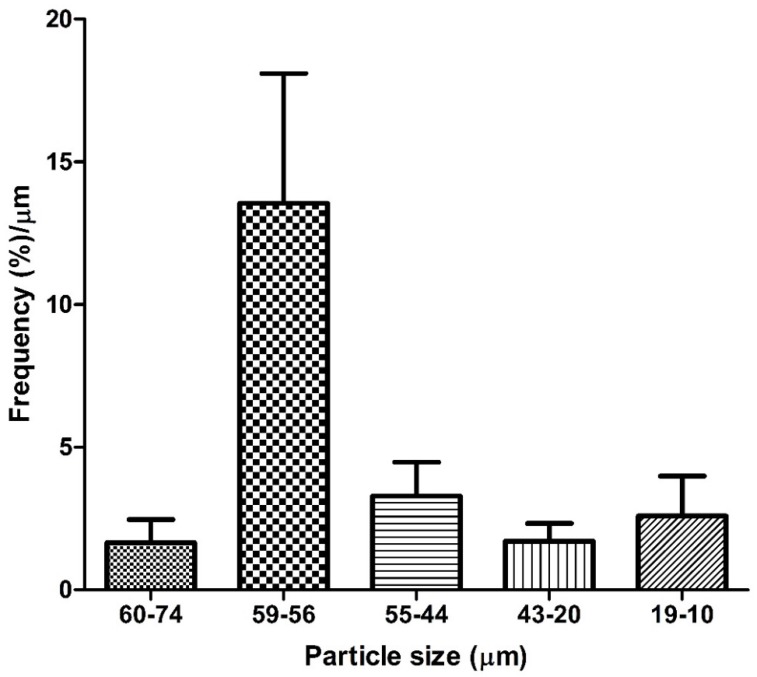
Distribution of particles removed from CAFs collected.

The size distribution analysis of particles removed from the CAFs is shown in Figure 5. These particles were mostly of a large size with equivalent diameters ranging from 74-10 µm. Particles within the range 74–60 µm were found in smaller amounts. They were lightweight, mainly waste of animal and vegetal origin like pieces of insects, leaves and wood (Figure 3). Nevertheless, particles with a size between 59–44 µm were the most abundant. Particles of 43–10 µm were found in smaller amounts.

### 3.1. Elemental Composition Was Analyzed by SEM with EDX.

The dust collected by the CAFs comes from Aguascalientes City, where the soil is classified as a semi-desert soil mainly composed of silicates and clay minerals, however, the dust collected by the CAFs displayed a more complex composition. The morphology and chemical composition of each particle was individually analyzed by SEM-EDX. The analysis of the dust particles separated from CAFs were composed of C, O, Si, Al, Ca, Mg, Na, K, Ti, and Fe. Si and Fe were the most abundant elements found in large particles; likewise, the particles display variable morphology. The shapes vary from rectangular to irregular, from spherical to triangular-like aggregates. 

**Figure 6:**
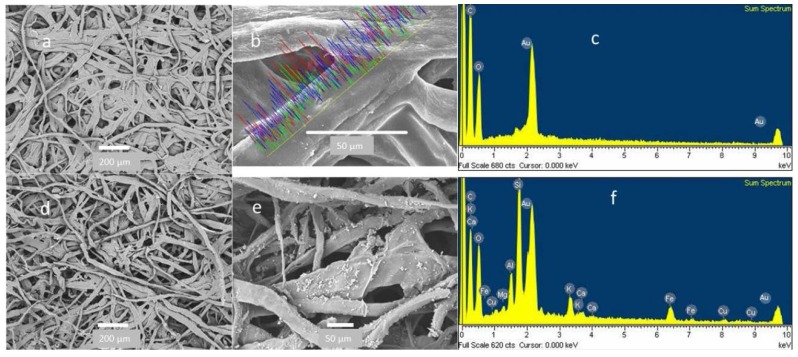
(**a–c**) Scanning electron micrographs and EDX spectrum of control filters and (**d–f**) collected CAFs.

The analysis of control CAFs showed a clean surface free of particles (Figure 6a,b) with fiber diameters of 7.5 ± 2.5 µm, n = 50, and cavities much larger than fiber diameters (15.28 ± 2.5 µm, n = 50). The elemental composition of control CAFs was 52.5% C and 47.5% O (Figure 6), meanwhile in the CAFs collected; the particles retained were different sizes; with equivalent diameter range from 16 to 0.7 µm (Figure 6d,f).

Based on the elemental composition and morphology results, these particles were sorted into three categories: mineral particles (derived from soil sediments and weathered rock surfaces), metallic particles (derived from industrial activities), and biogenic particles (pollen derived from plants, considered as potentially allergenic).

### 3.2. Mineral Particles

**Figure 7:**
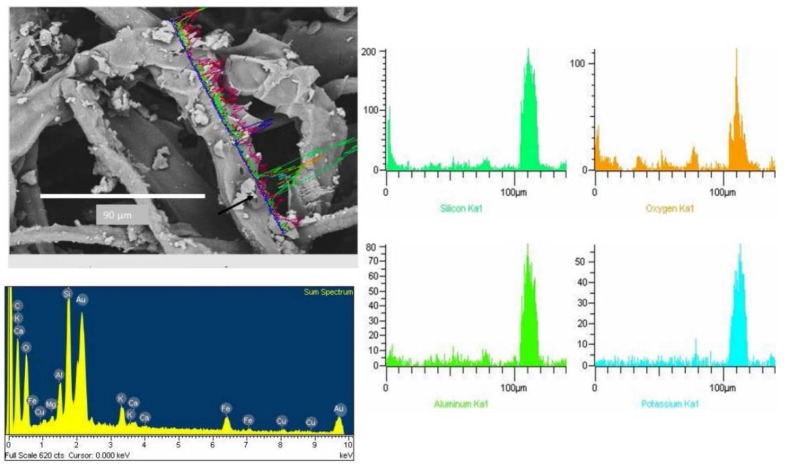
Identification of mineral K-feldspar particles by SEM-EDX (arrow particle <10 µm).

**Figure 8:**
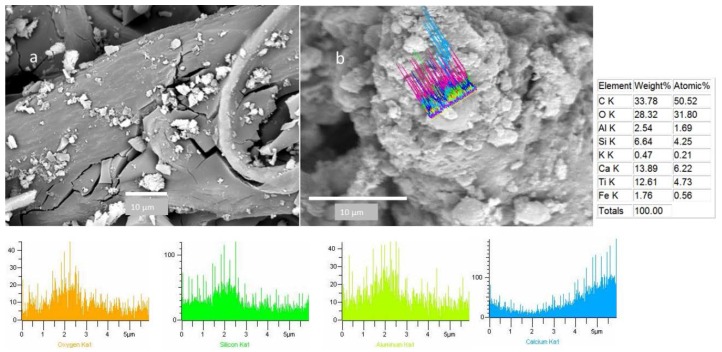
(**a**) Particles less than 2.5 µm and (**b**) nucleation and condensation of <2.5 µm particles that were deposited on CAF.

Most of the mineral particles observed on the CAFs had a diameter between 10-1.8 µm (Figure 7). This figure depicts the typical microphotography of an aluminosilicate particle, with a diameter <10 µm. Figure 8a shows particles with a diameter less than 2.5 µm (1.1 ± 0.5µm). SEM-EDX microphotograph data revealed different kinds of aluminosilicates with irregular morphology, which were comprised of Al, Si, and O, along with other minerals such as Ca, Fe, Mg, K and Ti. They were identified as feldspars (Figure 7 and Figure 8). Figure 7 also shows a collected CAF cellulose matrix with broken filter fibers and wider spaces between them, as would be expected of the filters used. The presence of particles deposited in the deepest fibers was also observed. By detailed analysis of the particles retained on the CAFs, it was noticed that some of them suffered a nucleation and condensation, especially among the smaller (2.5 µm), shown as aggregates (Figure 8b). 

### 3.3. Metallic Particles

**Figure 9:**
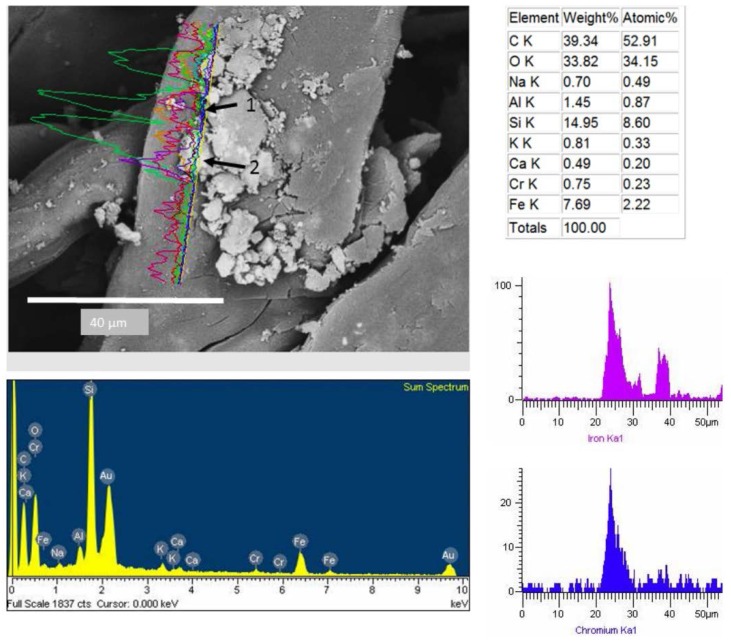
Metallic particles composed of (1) iron and (2) chromium.

**Figure 10:**
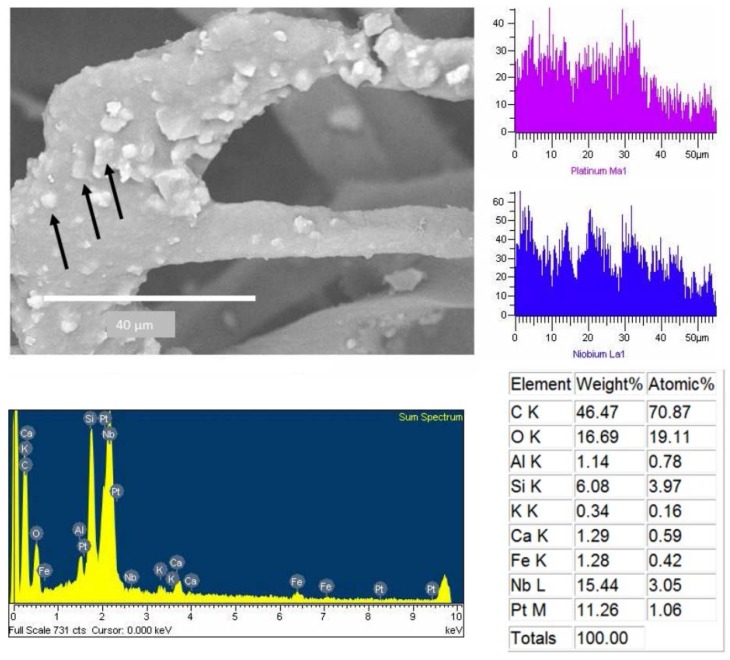
Metallic particle that contains platinum was found on CAF (black arrows).

The dust spectra analysis adhered to the CAFs revealed metallic particles (Figure 9 and Figure 10). Elemental mapping by EDX in one dimension recorded the elemental composition of two particles. The first with a diameter of 8 µm composed by Fe and Cr, whereas, the second has a small diameter of 3 µm with high content of Fe (Figure 9). 

**Figure 11:**
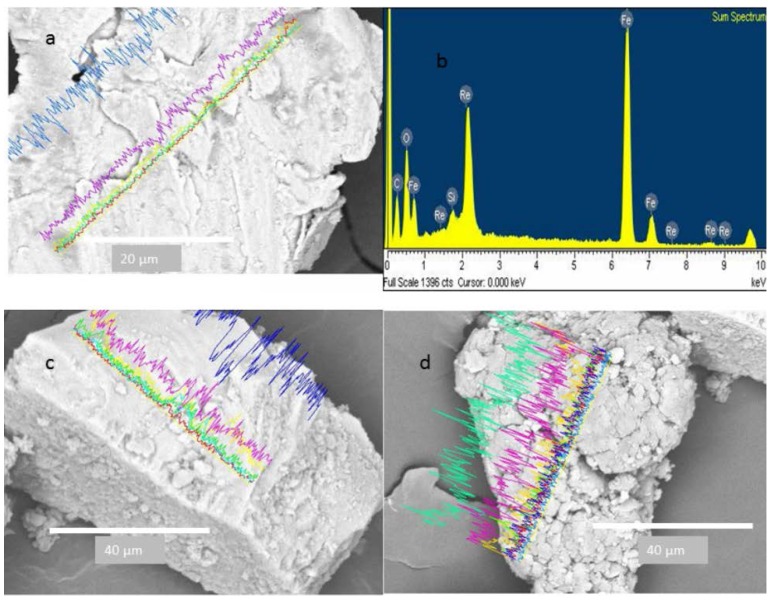
Iron particles with different morphologies. (**a**) Layer; (**b**) EDX spectrum; (**c**) rhomboid and (**d**) agglomerate.

Platinum was another element found in the adhered particles, which was observed in some particles even through the whole line mapping (Figure 10). Figure 11 shows the SEM of large metallic particles found at dust removed from the CAFs. The corresponding EDX spectrum revealed that these particles contain >70% iron (Figure 11b). These particles were identified as iron oxides composed of surface layers and with an amorphous shape (Figure 11a). Some of the particles had fine edges, which are characteristic of anthropogenic activity, with specific forms like spheres or rhomboid shapes and rough surfaces (Figure 11c,d). In the same way, it also shows small particles attached to the large metal particles. The particle in Figure 11d is decorated on its surface with many fine iron-rich particles smaller than a few micrometers.

### 3.4. Biological Particles

**Figure 12:**
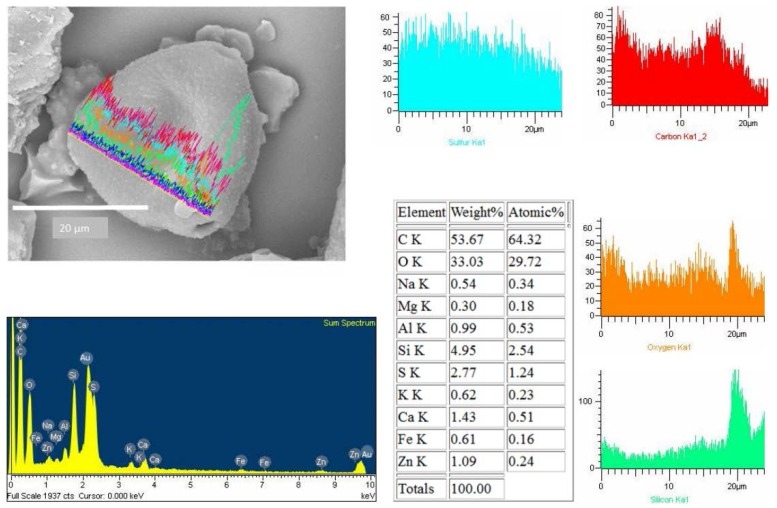
Pollen grain collected in Aguascalientes by CAFs.

**Figure 13:**
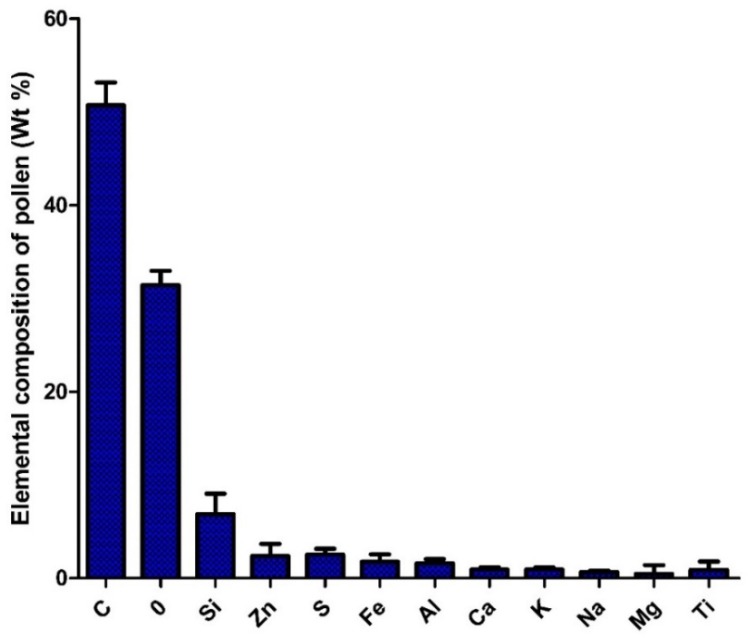
Frequency of elements in the composition of pollens collected by CAFs.

To obtain information about the morphology of pollen samples collected from the CAFs, dust samples were processed by acetolysis, and analyses of the images captured by optical microscopy and SEM were performed. A subfraction of 150 pollen grains were found in the analyzed dust of the 10 CAFs. Biological particles are typically identified by morphology and size range of 20 to 5 µm [17]. The pollen spectra was dominated by *Shinus molle, Lollium perenne, Prosopis sp*., *Eucalyptus*. Figure 12 shows a SEM image of pollen found in the CAFs. The pollen grain was found to be isopolar, radially symmetrical, non-angular, and 3-colporoidate, it was identified as a *Psidium guajava* pollen grain. The EDX results indicated that the main elemental composition of the pollen was C > O > Si > Zn > S > Fe > Al > Ca > K > Na > Ti (Figure 13). 

**Figure 14:**
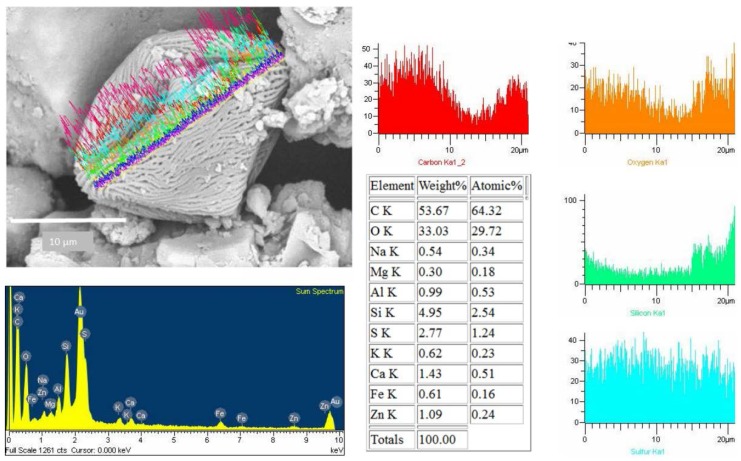
Pollen grain with adherent silicon particles collected by CAFs.

It’s interesting to note that all them, had Si and S in its composition. We used the linescan mode to determine the elemental composition of pollen that had a surface free of particles; however, some pollen samples that had PM on the surface were also analyzed using the linescan mode (Figure 14). This maneuver allowed us to study the association between the compositions of pollen and the adhered particles. The EDX spectra show that silicon is truly a constituent of pollen, and the particle on its surface also has a silicon oxide composition (Figure 12 and Figure 14).

## 4. Discussion

CAFs have been used for monitoring polychlorinated dibenzodioxins (PCDD) [12], polycyclic aromatic hydrocarbons (PAHs) [18], and heavy metals [19]; however, studies related to air particles have not yet been reported. We have now expanded this hypothesis and evaluated CAFs as active samplers of air pollution. A detailed characterization of individual atmospheric particles provided useful information about their sources, transport, and possible health effects. The particles retained by CAFs was less than the maximum capacity reported for CAFs (587 g) [20]. Perhaps, the reason for this result is that part of the retained particles fall into the box, as the CAFs are designed as impact filters with cellulose folds, so that when the vehicle is in motion, the particles of air entering the duct reach the same speed of the vehicle, resulting in collisions of the particles with the filter and fragmented particles will fall into the box with small particles being deposited between the folds. The impact of particles on the filter could change the distribution of sizes to more fine and ultrafine particles, favoring the deposition on the filter. Such particles can contribute to the group of particles detected in exhaust emissions [21]. Nonetheless, we determined the total area of filtration and estimated that the retained material was 0.78 g/m^2^, while, filtration efficiency was not determined under strict conditions, because the filters were not weighed previously and were collected after a distance was traveled. The estimate of the material amount collected by the filter allows us to assume that the filters studied were working at 100% and could eventually collect particles of all sizes as reported by Song et al. [8,22]. This value is in agreement with similar studies performed to collect powder from roads and avenues. One such study of road dust loadings reported values from 0.3 to 24.44 g/m^2^, with an average of 3.82 g/m^2^ [3]. Therefore, our reported value fits well with the road dust loadings. 

The nature of particles was inferred using light reflected microscopy, as these particles were easily identifiable by their reflective properties [23]. Animal and plant debris are frequently observed to be common elements of the aerosols collected from roads and highways [1,24,25]. The large metallic particles were found to have an initial source of waste removed by friction, vehicular, or anthropogenic activity. The multicolored inorganic particles were determined to be derived naturally from sands and small clays. A common observation was the presence of conglomerate asphalts [26]. This result was expected because Aguascalientes is a city with 100% paved streets. The constant friction of vehicles on the streets results in the release of small asphalt particles, which are then are impregnated with oil and PAHs [27].

The particles removed from the CAFs showed a distribution between 74 and 10 µm, and can thus be considered as large particles. The particles of 60–74 µm were mainly of plant and animal origin, and the particles between 74–10 µm were a mix of quartz, clay, metal and organic material composed mainly by pollen. Several studies have reported the large particles were the most abundant in dust from big cities in America and Asia [17]. Particles of 59–20 µm size were the most abundant, and they have been associated to a mineral origin and the transport of particles which are mixed with local powders from suburban areas to cities [3,17]. Similarly, CAF tests are often carried out with coarse Arizona dust, which has a size distribution predominantly in this range [28]. Particles smaller than 10 µm were less abundant in dust samples removed from CAFs, and rather were found in clusters with large particles and adhered to pollen grains.

Such particles were also observed adhered to the cellulose fibers CAFs (Figure 6, Figure 7, Figure 8, Figure 9 and Figure 10). These particles showed a diameter of between 16 and 0.7 µm, indicating that these filters are capable of retaining particles smaller than 2.5 µm. These results are comparable to published studies about vehicle cabin air inlet filters [29], somehow coinciding with the HEPA standards [15,26]. The design of automotive filters is based on their ability to filter particles, which is dependent on the mesh and the structure and composition of the fiber [30]. The analysis of CAFs showed spaces much larger than the fiber diameters, allowing substantial air flow, whereas fibers have a higher specific surface area. This arrangement promotes the deposition of particles with diameter less than 10 µm. These particles are deposited deep inside filters where they form agglomerates that decrease the filtration and the filter life [22]. The observation of agglomerates deep in the filter suggests that the separation method used was unable to remove all particles, and an alternative method must be used to estimate these particles. 

The EDX spectrum peaks correspond to the energy levels for carbon and oxygen. The carbon percentage is higher than the oxygen percentage, as expected for cellulose (C_6_H_10_O_5_)_n_. Since hydrogen cannot be detected by SEM-EDX, this element does not appear in the SEM-EDX spectrum [31]. Thus, we found from the elemental analysis that control CAFs possess only carbon, oxygen, and hydrogen. These typical characteristics of cellulose materials have been described previously [32].

Mineral particles were the most abundant and likely derived from natural sources, although there was also potential contribution of anthropogenic activities, such as construction, transport, and paving [33,34]. The elemental composition was mainly of Si, Al and O, identified as aluminosilicates corresponding to the Earth's crust. The soil particle size analysis classifies to these particles as silt and clays and suggest that due to their aerodynamic size and vehicle traffic, these particles can travel long distances. The topology of paved roads favors its deposition in the streets and avenues of the city. The winds and continuous movement of vehicles, which leads to recirculation of particles in the air, generates an “urban dust cycle” [2].

Iron is the most abundant element in metallic particles [1,35]. These particles were found in many shapes (Figure 11). Due to the formation of aggregates of small iron particles, which could disintegrate and solubilize, soluble iron oxide has been found to play an important role in the production of highly deleterious hydroxyl radicals, which are known to be a pathogenicity factor of smaller particle PM_10_ [36]. The presence of chromium and platinum in the particles could be associated with chrome-plating process in automotive parts. In particular, platinum is an element used in catalytic converters, which catalyze the conversion of nitrogen oxides into nitrogen and oxygen. Platinum is released from exhaust fumes and has been previously studied [37]. Palacios et al. [38] concluded that platinum is a principal component in exhaust fumes, with a particle size of less than 10 µm, and is released into airborne and dust sediments.

The detection of N and H was not possible under these measurement conditions, despite their presence in the filtered matter. It is known that pollen is mainly composed of organic compounds, including proteins, lipids, carbohydrates, vitamins, and hormones, but that pollen also contains minerals. C, H, O, and N are the main elements that constitute those organic compounds. The C+O account for >75% of the organic compounds, while Na, K, Fe, Al, Zn, and Ca were present in minor amounts. Such a composition has been was used to sort plant matter from the other PM [25,39,40]. Duque et al. [41] found that the main elements in control pollen were C, O, N, P, Ca, S, K, Mg, Si, Cl, and Na. Similar trace elements were found in *Platanus acerifolia* leaves [42].

Numerous studies have been reported on the elemental composition of PM deposited on pollen and leaves [43,44] as well as on their relationship to allergies [45]. Interestingly, some grains of pollen possess significant concentrations of silicon and sulfur on the surface [46,47,48]. The silicon come from natural origin, however, the presence of S could be related with a process of adsorption of noxious gases. 

The pollen spectra collected from the car air filters are typical species found in Aguascalientes State such as *Shinus molle, Lollium perenne* and *Prosopis sp.* Some of these plants are highly allergenic [49] and are widely distributed in the streets and gardens of Aguascalientes. The presence of *Psidium guajava* pollen in the samples analyzed from the CAFs, could be explained due some cars running in the Guajava Valley, a zone highly cultivated for exportation. A similar study performed by More et al. [50] showed that the use of CAFs as a pollen sampler was associated with the vehicle travel route. 

## 5. Conclusions

From reflected light microscopy and SEM- EDX studies, it follows that CAFs retain particulate matter of different nature, which could be classified into three main types: biological particles (material organic, plants and animal debris), mineral particles (clays and asphalt) and metal particles (composed mainly of Fe). For the study these particles were separated into two categories: those which they were removed from CAFs mechanically and those that remained attached to CAFs. The predominant particles were large particles that had a diameter between 59-20 µm. However, SEM revealed adhered particles smaller than a few micrometers. The particles adhered to the CAF fibers were between 16 to 0.7 µm. whereas, control filters showed a free surface of particles with a cellulose composition. These diverse groups of airborne particles had either natural or anthropogenic origin. The particle analyses provided insight into the possible source of these particles, mainly soil dust. The silicates were major contributors to the mineral particles. The metallic particles were composed of Fe, Cr, and Pt, and they were related to industrial activity, automotive parts and exhaust emissions. The palynological analysis of biological particles revealed the presence of pollen grains associated with the vegetation found along the high-traffic roads. These results allow us to say that car air filters capture a mixture of atmospheric particles, that can be analyzed to help monitor urban air.
